# Reduced fractional shortening of right ventricular outflow tract is associated with adverse outcomes in patients with left ventricular dysfunction

**DOI:** 10.1186/1476-7120-11-19

**Published:** 2013-06-03

**Authors:** Masashi Yamaguchi, Toshihiro Tsuruda, Yuki Watanabe, Hisamitsu Onitsuka, Kuniko Furukawa, Takeshi Ideguchi, Junji Kawagoe, Tetsunori Ishikawa, Johji Kato, Makoto Takenaga, Kazuo Kitamura

**Affiliations:** 1 Department of Internal Medicine, Circulatory and Body Fluid Regulation, Faculty of Medicine, University of Miyazaki, 5200 Kihara Kiyotake, Miyazaki 889-1692, Japan; 2 Internal Medicine, Fujimoto Central Hospital, 3584-1 Midarebashi Kitakawauchi, Miyazaki 880-0941, Japan; 3 Clinical laboratory, Miyazaki University Hospital, 5200 Kihara Kiyotake, Miyazaki 889-1692, Japan; 4 Frontier Science Research Center, University of Miyazaki, 5200 Kihara Kiyotake, Miyazaki 889-1692, Japan

**Keywords:** Heart failure, Right ventricle, Brain natriuretic peptide

## Abstract

**Background:**

Recent studies suggest the significance of right ventricular (RV) function in the outcome in patients with left ventricular dysfunction (LVSD); however, global assessment of RV remains to be determined by echocardiogram because of its complex geometry. This study aimed to validate RV outflow tract fractional shortening (RVOT-FS) in the evaluation of RV function and its prognostic value in patients with LVSD.

**Methods:**

This study included eighty-one patients (62 ± 17 years, mean ± SD, male 79%) with reduced LV ejection fraction (LVEF) (≤40%). Two-dimensional echocardiogram of the parasternal short axis view was obtained at the level of the aortic root, and RVOT-FS was calculated as the ratio of end-diastole minus end-systole dimension to end-diastole dimension.

**Results:**

RVOT-FS ranged from 0.04 to 0.8 (0.3 ± 0.2, mean ± SD), and correlated with LVEF (r = 0.33, p = 0.0028), RV fractional area change (r = 0.37, p = 0.0008) and brain natriuretic peptide level (r = -0.38, p = 0.0005). In Cox multivariate regression analysis, RVOT-FS [hazard ratio (HR) 0.028, 95% confidence interval (CI): 0.002-0.397]; p = 0.008] and New York Heart Association functional class III-IV [HR 2.233, 95% CI: 1.048-4.761]; p = 0.037] were independent factors to predict the events. During a median follow-up period of 319 days (1 to 1862 days), patients with RVOT-FS ≥ 0.2 showed a higher event-free rate than those < 0.2 by Kaplan-Meier analysis (log-rank test, p = 0.0016).

**Conclusions:**

Our data suggest that RVOT-FS is a simple parameter reflecting the severity of both ventricular function in patients with LVSD. In addition, RVOT-FS might be useful to predict adverse outcomes in such a patient population.

## Introduction

Left ventricular ejection fraction is well recognized in the adverse clinical outcomes of patients with chronic heart failure [1]; however, it loses statistical power when applied to patients with advanced heart failure [2]. In this setting, RV function determines exercise capacity and survival [2-4]. Although several echocardiographic parameters have been proposed [5], accurate global assessment of the RV is still challenging because of its complex anatomy; RV is not one chamber, but is composed of two distinct anatomic units, the RV sinus (from the tricuspid valve annulus to the proximal os infundibulum) and right ventricular outflow tract (RVOT) (from proximal os infundibulum to the pulmonary valve) [6]. Regional RVOT dysfunction is suggested to affect exercise tolerance after tetralogy of Fallot repair [7]. Thus, RVOT appears to have its own hemodynamic characteristics by reflecting the RV sinus and pulmonary artery [8]; however, few studies have addressed the impact of RVOT function on patients with left ventricular systolic dysfunction (LVSD). Based upon the unique nature of anatomy, we hypothesized that contractility of RVOT segment might be associated with the severity of heart failure and that it affects the prognosis of patients with LVSD. The aim of this study was to evaluate RVOT-fractional shortening (FS) with the clinical, laboratory and echocardiographic parameters. Second, we examined if the measurement of RVOT-FS is a useful parameter providing predictive value in these patients.

## Methods

### Patients

This study included 81 patients hospitalized at Miyazaki University Hospital between March 2007 and November 2011. Patients were selected based on the impaired LV systolic function (LV ejection fraction (EF) <40%) but not on clinical symptoms, and excluded if they had LVSD due to acute myocarditis and tachycardia-induced cardiomyopathy. Patients underwent coronary angiography, ^201^Tallium-/ ^123^I-β-methyl iodophenyl pentadecanoic acid scintigraphy, ^18^fluoro-deoxyglucose by positron emission tomography imaging, magnetic resonance imaging and endmyocardial biopsy to aid in the definition of the etiology of LVSD, such as ischemic heart disease, hypertensive heart disease, valvular heart disease and secondary cardiomyopathies (ex. sarcoidosis or other infiltrative cardiomyopathies). We notified the patients in writing and on the homepage, or gave written informed consent. This study was approved by the Human Investigation Review Committee of the University of Miyazaki (No.979) and conformed with the principles outlined in the Declaration of Helsinki (*Cardiovasc Res* 1997; 35: 2-4).

### Echocardiography

**Figure 1:**
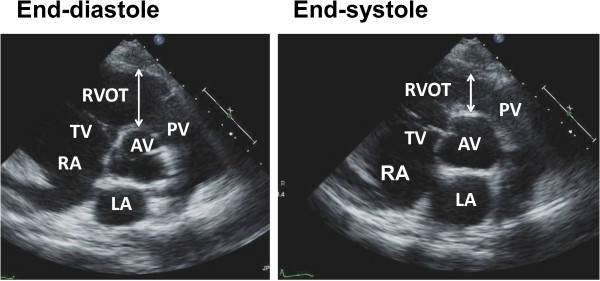
**Two-dimensional view of right ventricular outflow tract at end-diastole and end-systole.** RVOT, right ventricular outflow tract; PV, pulmonary valve; TV, tricuspid valve; RA, right atrium; LA, left atrium; AV, aortic valve.

Echocardiography was performed using an ATL Philips IE33 Ultrasound machine (Garnerville, NY, USA) with the determination of standard and Doppler parameters. Echocardiographic data on admission and at the out-patient clinic were stored (DICOM format), and reviewed digitally off-line on a desktop computer with ProSolv software (ProSolv Cardiovascular Analyzer 3.5; Indianapolis, IN, USA) by two experienced cardiologists. Size of the left atrium (LA), LV at end-diastole (LVDd) and end-systole (LVDs), and wall thickness of the intraventricular septum (IVSTd) and posterior wall (LVPWTd) at diastole were determined from the two-dimensional parasternal long-axis view on electrocardiogram. LVEF was calculated by the modified Simpson’s method in apical four- and two-chamber views. For evaluating the RV fractional area change (RVFAC), endocardial borders were outlined at end-diastole and end-systole, using an apical four-chamber view [9], and RVFAC (%) was calculated by (RV end-diastolic area – RV end-systolic area)/RV end-diastolic area x100. Tricuspid regurgitation-pressure gradient (TR-PG) was estimated by the peak continuous-wave Doppler velocity of the tricuspid regurgitation jet using the modified Bernoulli equation. The RVOT dimension was measured in end-diastole and end-systole at parasternal-short axis view at the level of the aortic root using two-dimensional echocardiogram between 1 to 3 cardiac cycles on electrocardiogram, and RVOT-FS was calculated as (dimension at end-diastole – end-systole)/ end-diastole (Figure 1). This was modified to the original study by Lindqvist et al. [8] who determined the RVOT dimension on M-mode. Each patient was asked to return for a follow-up echocardiogram or it was performed when they were re-admitted to the hospital.

### Cine magnetic resonance imaging (cine MRI)

We validated the RVOT-FS assessed by echocardiogram with 1.5-Tesla MRI system (Signa HDxt, GE Healthcare UK Ltd., Buckinghamshire, United Kingdom). Nine subjects were in the supine position with breath holding in expiration and with a vector-cardiographic method for electrocardiographic gating. Transaxial slices using the rephrased gradient echo technique was planned to cover the heart from a level just below of diaphragm to bronchial bifurcation, with repetition time of 2.7-2.9 msec, echo time of 0.8-1.0 msec, a flip angle of 40 degree, and a slice thickness of 10 mm. The diameters of RVOT at end-diastole and end-systole were measured on still frames with manual tracing by means of a track-ball cursor.

### Laboratory tests

Plasma brain natriuretic peptide (BNP) concentration was measured by chemiluminescent immunoassay (ARCHITECT BNP-JP; Abott, Chiba, Japan). C-reactive protein in the serum was determined by the latex agglutination test with a measurement range of 0.01 to 32 mg/dL. Estimated glomerular filtration rate (eGFR) was calculated using the equation for the Modification of Diet in Renal Disease [10].

### Follow-up and end points

Follow-up was performed by direct examination of medical records or by contact with cardiologists. All patients were followed up until October 2012 unless a major end point terminated the follow-up. In the present study, outcomes were included such as death from cardiovascular diseases (heart failure and lethal arrhythmia), requirement of cardiac transplantation, and unexpected hospitalization due to the worsening of heart failure [11].

### Statistical analysis

Data analyses were performed using GraphPad Software Prism 5 (GraphPad Software, San Diego, CA, USA) for Bland-Altman plot and SPSS version 11.0 (SPSS Japan, Tokyo, Japan) for the rest of the analyses. We used Student’s *t*-test or Mann-Whitney test for two continuous parametric or nonparametric variables, and the Fisher’s exact test for qualitative data such as numbers and percentages. Unadjusted and adjusted associations of RVOT-FS with other clinical parameters were evaluated by Spearman rank-correlation coefficient and multivariate linear regression. There were close relationships between the following two variables [LVDd and LVDs (r = 0.886, p < 0.0001)] and [IVSTd and LVPWTd (r = 0.650, p < 0.0001)], and we removed LVDs and LVPWTd as variables in the analysis. A Cox proportional hazards model was used to determine any variables as significant factors to predict cardiovascular events in the study subjects. The covariates included in this model were age, sex, use of β-blocker, angiotensin converting enzyme inhibitor (ACE-I) /angiotensin II type 1 receptor antagonist (ARB), calcium channel blocker, diuretic and aldosterone antagonist, rhythm in electrocardiogram, echocardiographic parameters (LAD, IVSTd, LVDd, LVEF, RVFAC, RVOT-FS, TR-PG), BNP, eGFR, hemoglobin, total bilirubin, uric acid, C-reactive protein and New York Heart Association (NYHA) functional class. Results are presented as relative risk with 95% confident interval (CI). Kaplan-Meier analysis was constructed by receiver operating characteristics (ROC) analysis. In addition, Bland-Altman plots were used to compare the agreement of the two methods (echocardiography vs. cine MRI). The plot was displayed as the average of the two values and the difference between the two measurements. Results are shown as absolute numbers (%) or the mean ± standard deviation (SD). Statistical significance was accepted when p < 0.05.

## Results

### Patients’ characteristics

Table 1 summarizes clinical characteristics and echocardiographic variables of the 81 patients enrolled in this study. Etiology of LVSD was heterogeneous, and 23% of them exhibited atrial fibrillation, and mean value of LVEF was 30%.

**Table 1:** Patient’s characteristics

Age, years	62 ± 17
Sex	Male (79%)
NYHA functional class III-IV	17 (21%)
(I 31; II 33; III 15; IV 2)	
Etiology	
IHD	22 (27%)
Non-IHD	59 (73%)
Valvular disease	4 (5%)
DCM	37 (46%)
HCM	5 (6%)
HHD	7 (9%)
Others	6 (7%)
Electrocardiogram	
Sinus rhythm	54 (67%)
Atrial fibrillation	19 (23%)
Pacing rhythm	8 (10%)
Medications	
Beta-blocker	62 (78%)
ACE-I/ARB	65 (80%)
Calcium channel blocker	16 (20%)
Diuretic	61 (75%)
Aldosterone antagonist	47 (58%)
Laboratory test	
BNP (pg/mL)	765 ± 870
Uric acid (mg/dL)	7.4 ± 2.6
Hemoglobin (g/dL)	14.0 ± 2.1
Total bilirubin (mg/dL)	1.1 ± 0.8
C-reactive protein (mg/dL)	1.5 ± 2.9
eGFR (mL/min/1.73 m^2^)	51 ± 20
Echocardiographic examination	
LAD (cm)	4.1 ± 0.8
IVSTd (cm)	1.0 ± 0.3
LVPWTd (cm)	1.1 ± 0.3
LVDd (cm)	6.1 ± 1.0
LVDs (cm)	5.4 ± 1.1
LVEF (%)	30 ± 8
RVFAC (%)	35 ± 14
TR-PG (mmHg)	25 ± 12

Values are expressed as absolute numbers (%) or the mean ± SD.

*NYHA* New York Heart Association, *IHD* ischemic heart disease, *DCM* dilated cardiomyopathy, *HCM* hypertrophic cardiomyopathy, *HHD* hypertensive heart disease, Others include 2 sarcoidosis, 1 LV noncompaction, 1 restrictive cardiomyopathy and 2 unknown origin, *ACE-I* angiotensin converting enzyme inhibitor, *ARB* angiotensin II type 1 receptor antagonist, *BNP* brain natriuretic peptide, *eGFR* estimated glomerular filtration rate, *LAD* left atrial dimension (diastole), *IVSd* intraventricular septal wall thickness (diastole), *LVPWTd* left ventricular posterior wall thickness (diastole), *LVEF* left ventricular ejection fraction, *RVFAC* right ventricular fractional area change, *TR-PG* tricuspid regurgitation-pressure gradient.

### Spectrum of RVOT-FS

**Figure 2:**
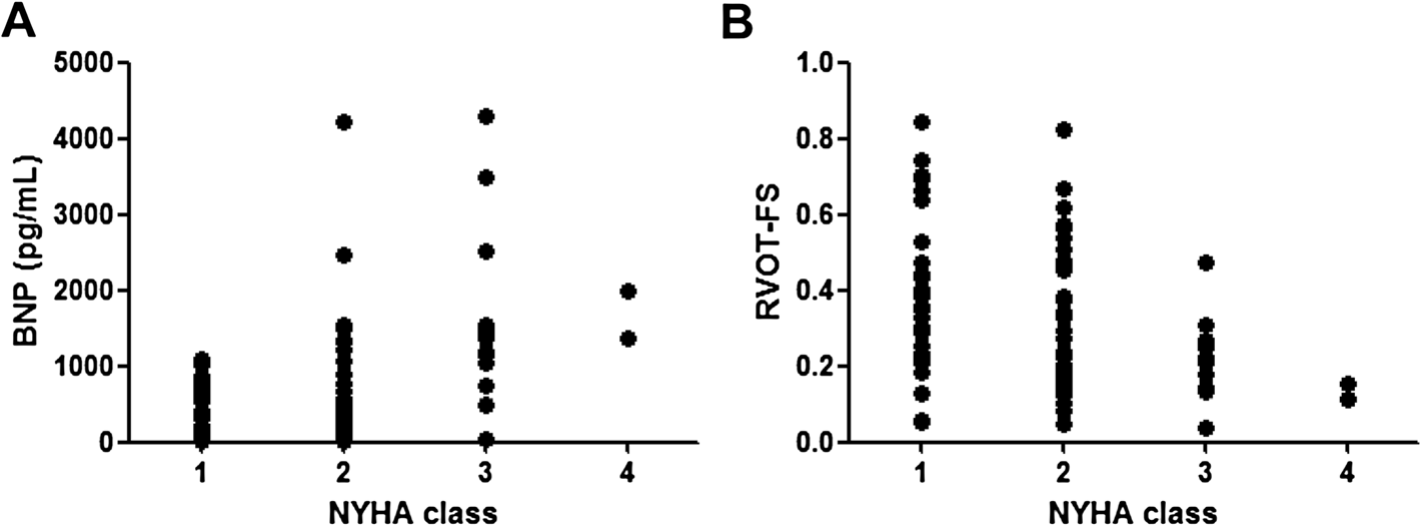
**Plasma BNP level (A) and RVOT-FS (B), according to NYHA functional class in LVSD patients.** Number of patients in NYHA functional class were I 31; II 33; III 15; IV 2.

RVOT diameter was 3.1 cm at end-diastole (range, 1.9-5.3 cm) and 2.1 cm at end-systole (range, 0.4-4.5 cm), and the mean value of RVOT-FS was 0.3 (range, 0.04-0.8). Table 2 summarizes that RVOT-FS was associated with NYHA functional class III-IV (r = -0.27), LAD (r = -0.45), LVDd (r = -0.29), LVDs (r = -0.32), LVEF (r = 0.33), RVFAC (r = 0.37), BNP (r = -0.38), uric acid (r = -0.28), total bilirubin (r = -0.30), or C-reactive protein (r = -0.29), but not with age, IVSTd, LVPWTd, TR-PG or eGFR. Figure 2 shows that plasma BNP level increased (A), whereas RVOT-FS decreased (B), according to NHYA functional class. As shown in Table 3, RVOT-FS was independently associated with BNP and LVDd.

**Table 2:** Correlation of RVOT-FS with clinical and echocardiographic variables

	**r**	**p value**
Age	0.14	0.2282
NYHA III-IV	-0.27	0.0132
LAD	-0.45	<0.001
IVSTd	0.05	0.656
LVPWTd	0.13	0.258
LVDd	-0.29	0.01
LVDs	-0.32	0.003
LVEF	0.33	0.0028
RVFAC	0.37	0.0008
TR-PG	-0.21	0.0963
BNP	-0.38	0.0005
Uric acid	-0.28	0.0116
Total bilirubin	-0.30	0.0061
C-reactive protein	-0.29	0.0092
eGFR	0.20	0.086

*NYHA* New York Heart Association, *LAD* left atrial dimension (diastole), *IVSTd* intraventricular septal wall thickness (diastole), *LVPWTd* left ventricular posterior wall thickness (diastole), *LVDd* left ventricular end-diastolic dimension, *LVDs* left ventricular end-systolic dimension, *LVEF* left ventricular ejection fraction, *RVFAC* right ventricular fractional area change, *TR-PG* tricuspid regurgitation-pressure gradient, *BNP* brain natriuretic peptide, *eGFR* estimated glomerular filtration rate.

**Table 3:** Multivariate regression analysis for RVOT-FS

**Dependent variable**	**Independent variable**	**Regression co-efficient**	**p value**
RVOT-FS	BNP	-0.415	0.001
	LVDd	-0.324	0.005

Age, NYHA functional class, LAD, IVSTd, LVDd, LVEF, RVFAC, TR-PG, total bilirubin, uric acid, BNP, C-reactive protein and eGFR were included as independent variables.

### Clinical characteristics of patients with or without cardiovascular event

During a median follow-up period of 319 days (1 to 1862 days), all-cause events occurred in 38 patients (cardiac death, 13; requirement of cardiac transplantation, 2; unexpected hospitalization due to the worsening of heart failure, 23). Table 4 summarizes that patients who had an event were older, with higher BNP and total bilirubin level, and a greater prevalence of diuretic use. On the other hand, they showed a lower concentration of eGFR. On the echocardiogram, LAD was significantly increased, whereas RVOT-FS and IVSTd were decreased in the event group. LVEF, RVFAC or TR-PG did not change between the two groups.

**Table 4:** Clinical characteristics and echocardiographic variables of patients with or without cardiovascular event

**Parameters**	**(-) (n = 43)**	**Cardiovascular event**		
		**(+) (n = 38)**	**p value**	
Age	58 ± 17	66 ± 15	0.037	1)
Male	36	28	0.289	2)
NYHA functional class III-IV	6	12	0.067	2)
IHD	10	10	0.800	2)
Rhythm (Af / pacing)	10	17	0.059	2)
BNP (pg/mL)	580 ± 805	965 ± 903	0.005	3)
Total bilirubin (mg/dL)	0.93 ± 0.53	1.34 ± 0.98	0.018	1)
Uric acid (mg/dL)	7.08 ± 1.89	7.76 ± 3.18	0.235	1)
eGFR (mL/min/1.73 m^2^)	55.8 ± 18.9	44.9 ± 19.8	0.015	1)
Hemoglobin (g/dL)	14.3 ± 2.3	13.5 ± 1.9	0.081	1)
C-reactive protein (mg/dL)	1.4 ± 3.0	1.5 ± 2.9	0.039	3)
LAD (cm)	3.89 ± 0.66	4.43 ± 0.91	0.003	1)
IVSTd (cm)	1.08 ± 0.35	0.87 ± 0.24	0.004	1)
LVPWTd (cm)	1.11 ± 0.27	1.01 ± 0.21	0.070	1)
LVDd (cm)	6.06 ± 0.79	6.22 ± 1.25	0.465	1)
LVDs (cm)	5.28 ± 0.85	5.50 ± 1.28	0.356	1)
LVEF (%)	31.4 ± 7.6	28.5 ± 7.8	0.093	1)
RVOT-FS	0.38 ± 0.20	0.27 ± 0.17	0.008	1)
RVFAC (%)	38 ± 14	32 ± 14	0.086	1)
TR-PG (mmHg)	25 ± 14	25 ± 9	0.978	1)
Beta-blocker	25	29	0.102	2)
ACE-I/ARB	28	30	0.219	2)
Calcium channel blocker	11	6	0.413	2)
Diuretic	24	36	<0.0001	2)
Aldosterone antagonist	21	25	0.177	2)

Values are expressed as absolute number or the mean ± SD. Each variable was analyzed by 1) unpaired t-test, 2) Fisher's exact test and 3) Mann-Whitney test.

*NYHA* New York Heart Association, *IHD* ischemic heart disease, *Af* atrial fibrillation, *BNP* brain natriuretic peptide, *eGFR* estimated glomerular filtration rate, *LA* left atrial dimension (diastole), *IVSTd* intraventricular septal wall thickness (diastole), *LVPWTd* left ventricular posterior wall thickness (diastole), *LVDd* left ventricular end-diastolic dimension, *LVDs* left ventricular end-systolic dimension, *LVEF* left ventricular ejection fraction, *RVFAC* right ventricular fractional area change, *TR-PG* tricuspid regurgitation-pressure gradient, *ACE-I* angiotensin converting enzyme inhibitor, *ARB* angiotensin II type 1 receptor antagonist.

### Univariate and multivariate Cox regression analyses

**Figure 3:**
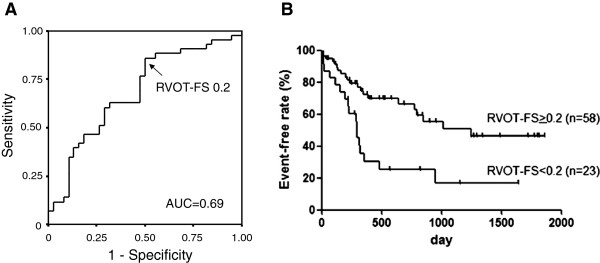
**A, Receiver operating characteristics (ROC) analysis and B, Kaplan-Meier analysis to evaluate the cardiovascular event-free rate according to the RVOT-FS in LVSD patients.** AUC, area under the curve.

Table 5 summarizes the univariate and multivariate Cox regression analyses using forward step-wise variable selection. In univariate regression analysis, RVOT-FS, NYHA functional class III-IV, IVSTd, diuretic use, BNP and total bilirubin were extracted as significant factors for predicting cardiovascular events. In multivariate Cox regression analysis, RVOT-FS and NYHA functional class III-IV remained independent factors. Based on the ROC analysis with sensitivity of 86% and specificity of 47% (Figure 3A), we divided the patients into RVOT-FS > 0.2 or < 0.2. As shown in Figure 3B, RVOT-FS > 0.2 had a higher prevalence of event-free periods, compared with < 0.2 (log-rank test p = 0.0016).

**Table 5:** Univariate and multivariate Cox regression analyses

**Univariate analysis**				**Multivariate analysis**		
**Variables**	**HR**	**95% CI**	**p value**	**HR**	**95% CI**	**p value**
RVOT-FS	0.110	0.014-0.886	0.038	0.028	0.002-0.397	0.008
NYHA functional class III-IV	2.828	1.380-5.793	0.004	2.233	1.048-4.761	0.037
IVSTd	0.112	0.030-0.415	0.001			
Diuretic use	6.872	1.654-28.548	0.008			
BNP	1.000	1.000-1.001	0.018			
Total bilirubin	1.675	1.217-2.306	0.002			

Univariate and multivariate Cox regression analyses were constructed with forward step-wise variable selection. *HR* hazard ratio, *CI* confidence interval. Covariates included age (year), sex (male, 1; female, 2); NYHA functional class III-IV (0, I-II; 1, III-IV); sinus rhythm in electrocardiogram (no, 0; yes, 1); BNP (pg/mL), eGFR (mL/min/1.73 m^2^), hemoglobin (g/dL), total bilirubin (mg/dL), uric acid (mg/dL) and C-reactive protein (mg/dL), echocardiographic parameters such as LAD (cm), IVSTd (cm), LVDd (cm), LVEF (%), RVFAC (%), RVOT-FS, TR-PG (mmHg); use (no, 0; yes, 1) of beta-blocker, ACE-I/ARB, calcium channel blocker, diuretic and aldosterone antagonist.

### Reproducibility, and validation of RVOT-FS with cine MRI

**Figure 4:**
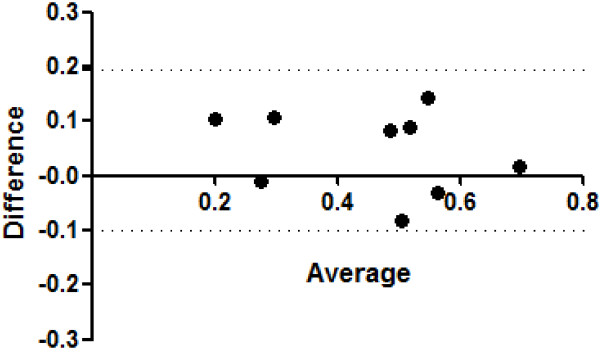
**Bland-Altman plots of RVOT-FS measurement between echocardiography and cine MRI to determine the substantial variability of fixed or proportional bias.** The 95% limits of agreement are shown as two dotted lines.

Reproducibility of the RVOT-FS measurement was evaluated by calculating the intra- and inter-observer variability in 20 patients. The intra- and inter-observer coefficient variables were 2.5 and 14.7, respectively. We also carried out cine MRI for nine subjects to examine the validity of RVOT-FS assessed by echocardiogram. The Bland-Altman plots demonstrated no evidence of substantial fixed or proportional bias in the two methods (Figure 4).

## Discussion

Assessment of RV function is important to understand the pathophysiology of heart failure; however, it is still challenging to find a simple and comprehensive parameter by echocardiogram. In this study, we demonstrated that RVOT-FS reflects the severity of both left- and right-sided ventricular function. In addition, our data suggested that RVOT-FS might be a useful parameter to predict cardiovascular events in patients with LVSD.

RV originates from a different embryological source to LV [12]. RVOT is defined as a region between the sub-pulmonary infundibulm and pulmonary valve, and is distinct from the rest of RV in origin and anatomy [5,6,8]. The measurement of RVOT has not been standardized in healthy subjects; however, the mean diastolic dimension is reported to be 2.8 cm [5] and RVOT-FS is 0.61 [8] to 0.98 [13]. In this study, patients with LVSD exhibited a relatively enlarged diastolic dimension of RVOT along with its reduced contractility. The pathophysiological and clinical relevance of RVOT-FS measurement was not completely elucidated in this study; however, several potential mechanisms can be suggested. Original study by Lindqvist et al. [8] showed that RVOT-FS correlated with several RV functional parameters, and our data support that RVOT-FS positively correlated with RVFAC. There are few reports to demonstrate the RVOT-FS with clinical, laboratory and echocardiographic variables, and our data indicate the unique characteristics of this value. Positive correlation with total bilirubin concentration is likely to reflect the increased central venous pressure by the impaired RV hemodynamics [14], and inverse correlation with serum concentrations of uric acid [15] and C-reactive protein [16], in part by indirectly reflecting the chronic systemic inflammation, oxidative stress and diuretic use. It is of note that RVOT-FS was also associated with chamber size of LA and LV, and LVEF. We found that RVOT-FS is associated with LV end-diastolic dimension and BNP independently, suggesting that the magnitude of RVOT-FS reflects the structural and functional capacity of LV as well as RV [17,18]. The continuity of superficial muscle fibers encircle RV and LV represents the traction of both ventricular free walls, and makes up the anatomic basis for mechanical ventricular-ventricular interaction [19,20]. LV hemodynamic behavior was reported to be indirectly assessed by the motion of aortic root [21], and our data implicate that LV overload influences the motion of RVOT. In other point of view, we speculate that the regional contractility of RVOT may affect the LV stroke volume if LV function have failed, because the RVOT segment contributes to up to 15% of total RV stroke volume [6], but need further investigation. In line with previous reports [14,22-25], cardiovascular events occurred in patients who were older, with the increased LA size, BNP and total bilirubin concentration, and frequent diuretic use. Interestingly, the prevalence of events was greater in patients with decreased RVOT-FS than LVEF, TR-PG or RVFAC. A number of factors contribute to the outcome in patients with advanced heart failure. In this study, diuretic use, BNP and total bilirubin, LV wall thickness as well as RVOT-FS and NYHA functional class was extracted as the predictors of follow-up patients by Univariate Cox regression analysis. LV overload is initially compensated by the adequate increase of wall thickness, and our data support that the reduced LV wall thickness exhibited poor outcome, and it appears to be a consequence of maladaptive LV remodeling resulting from myocyte cell loss [26]. Multivariate Cox regression analysis demonstrated that RVOT-FS as well as the severity of heart failure was an independent prognostic value, and we showed that RVOT-FS <0.2 exhibited poorer prognosis. However, the specificity of cut-off value chosen by ROC curve was low, and this might have been due to the wide distribution of RVOT-FS in patients with NYHA functional class I or II. In the follow-up echocardiogram, we observed that deterioration of RVOT-FS with a minimal change in LVEF resulted in a poor outcome (data not shown). Drighil et al. [13] reported that RVOT-FS improved more rapidly than other RV functional parameters such as RVFAC or tricuspid annular plane systolic excursion after mitral valve commissurotomy. Thus, sequential assessment of RVOT-FS by echocardiogram might lead to a more accurate diagnosis and would help to determine the medical approach. Furthermore, it would be interesting whether the intervention of any specific pharmacotherapy and medical device to improve the cardiovascular mortality/morbidity are associated with the contractility of RVOT.

### Study limitation

This is a retrospective observation study with a small number of heterogeneous pathologies in patients with LVSD. We did not assess the other RV systolic parameters, such as peak systolic tricuspid annular velocity, integral of the systolic wave, and tricuspid annular plane systolic excursion [27]. In addition, we understand that an oblique section of echocardiographic imaging at the level of RVOT leads to underestimation of the value. Moreover, this study included 23% patients with atrial fibrillation, and single measurement of RVOT-FS potentially hampers the accurate value. We validated the value with cine MRI, but we agree that the reproducibility was sub-optimal [28], and it is necessary to improve it by newer technologies [20].

## Conclusion

The present study suggests that RVOT-FS is a parameter to assess the severity of both ventricular functions in LVSD patients. In addition, our findings implicate a measurement of RVOT-FS might provide a predictive value in such a patient population.

## Abbreviations

RVOT: Right ventricular outflow tract; LVSD: Left ventricular systolic dysfunction; FS: Fractional shortening; EF: Ejection fraction; LA: Left atrium; LVDd: LV dimension at end-diastole; LVDs: LV dimension at end-systole; IVSTd: Wall thickness of the intraventricular septum; LVPWTd: Wall thickness of the posterior wall; TR-PG: Tricuspid regurgitation-pressure gradient; RVFAC: RV fractional area change; MRI: Magnetic resonance imaging; BNP: Brain natriuretic peptide; eGFR: Estimated glomerular filtration rate; ACE-I: Angiotensin converting enzyme inhibitor; ARB: Angiotensin II type 1 receptor antagonist; NYHA: New York Heart Association; ROC: Receiver operating characteristics; SD: Standard deviation.

## Competing interests

The authors have no competing interest.

## Authors' contributions

MY, TT and KK designed the study, investigated all patients and analyzed the data. MY and TT wrote the manuscript. MY, TT and KF performed echocardiogram and acquired the measurement. YW and MT performed cine MRI and analyzed the data. HO, T. Ideguchi, J. Kawagoe, and T. Ishikawa have interpreted the data and have been involved in drafting the manuscript. J. Kato performed the statistical analysis and made critical review of the manuscript. All authors have read and given final approval of the manuscript.
